# Notes and Comments

**Published:** 1923-10

**Authors:** 


### NOTES AND COMMENTS.

### THE GROWTH OF CANCER.

SOME strange contrasts between our success and our failure in dealing with disease are brought out by Sir George Newman's report on the health of 1922. We have learned how to confine outbreaks of smallpox within manageable proportions; tuberculosis is diminishing; and notifications of typhoid are not much more than one-seventh of the number in 1911. On the other hand, we have the terrible facts that the death-rate from cancer is more than seven times what it was when Queen Victoria came to the throne, and that it is always increasing. Between the first year of the century and 1921 the rate per million went up nearly 50 per cent. The increase is partly, but only partly, accounted for by better diagnosis—the growth of 25 per cent. since 1911 most certainly cannot be accounted for in that way. Should such rapid progression continue we shall, before long, be recording thousands of deaths per million from cancer instead of the 1,215, heavy as that rate is, of the year before last. Hitherto cancer research has completely failed, and the world awaits, with ever-growing anxiety, the beam of light which must some day illumine the cause of this dread disease and the path which leads to its cure.

### STILL C3.

"THE general standard of men presenting themselves for enlistment has been poor." So we read in the annual report on the British Army for the year ending September 30, 1921, which, with an absence of fuss and scurry which is delightful in these hurrying days, has just been issued. The sentence we have quoted seems mild enough when we turn to the figures and find that almost exactly one-half of the men who offered themselves for enlistment were rejected as physically unfit. The percentage of rejections in the London zone actually exceeded 56, and was only a fraction less in the Western zone; fitness was much more marked in Ireland than elsewhere—even in Scotland, where it used to be supposed that oatmeal created a peculiarly hardy type, there were more than 48 per cent. of rejections. The authors of the report find comfort in the explanation that many of those who offered themselves were growing lads during the hardships of the war, and that a gradual improvement is now going on. This does not account for the comparatively low physical standard which was revealed by the war, nor for the fact that the educational attainments of the recruits are less than before that time. This melancholy story of weak bodies and stunted mentalities suggests that, at the best, a generation must elapse before we emerge from the ranks of C3 nations.

### CONTRIBUTORY SCHEMES.

THE summary of the position and progress of Contributory Schemes, issued by the Voluntary Hospitals Commission, from which we print considerable extracts on another page, is a highly interesting and satisfactory document. Experience has made it perfectly clear that we have been right in our persistent contention that to organise income, and to widen its sources, is not to dry up the springs of charity. The Contributory Schemes are now well established, and we may expect that, as time goes on, they will not only increase in number, but will receive extensions of their scope. The Commissions' Memorandum shows that there are now 73 schemes covering 102 hospitals, and that, although they vary with local conditions, they are all founded on the same principle. Deductions from wages are common to almost all. The employers, whose aid, with the consent of their staffs, is of course essential, include a farmer who posts the sum collected from his men. "A very small minority" of schemes, it is stated, attempt to guarantee some kind of insurance benefit, but the charitable instinct, and the recognition of the primary need of the sick poor, is the constructive principle behind them. Representation of the contributing workpeople is general. Had it not been, we may be sure that success would have been relatively small.

### THE SLUM.

SCARCITY of dwelling-houses has not only worked hardship to individuals, but has unquestionably caused serious injury to the public health, often in ways that are not immediately obvious. In particular, the scarcity of new houses has compelled many local authorities to tolerate slum areas which, in happier circumstances, would have been cleared away at any time since the trouble began in 1910. Happily we are no longer quite standing still. We learn with satisfaction from the annual report of the Ministry of Health that local authorities have been invited to submit schemes on the basis of Government grants to cover half the estimated annual loss, and that more than eighty Councils are preparing such schemes. This is merely to touch the fringe of a great and pressing subject, and we may hope that as the housing difficulty grows less it will become practicable to make much more rapid progress. The demolition of slums is at present not merely a matter of money. We have to find shelter for the dispossessed, which is extremely difficult, and until we can do so there will be little hope of improving the health and reducing the death-rate of very many of our towns, large and small. Meanwhile, scores of Medical Officers are compelled to look on helplessly while disease makes its ravages in crowded and germ-impregnated courts and tenements.

### COMPULSORY VACCINATION.

SO far we have not seen it suggested that the few cases of smallpox which have just been reported in London are merely chicken-pox, wrongly diagnosed. That suggestion is still being made in relation to the Gloucester outbreak, now happily at an end. It is impossible not to derive a certain amused satisfaction from the eagerness of many people to be vaccinated themselves, and to have their children inoculated, immediately there is a smallpox epidemic in their neighbourhood, or the threat of one. Thus each visitation substantially increases the number of persons protected from smallpox, but we are still a deplorably unvaccinated country, thanks to the weakness of Parliament and the readiness of magistrates to grant exemption certificates. The time has, indeed, come when vaccination ought again to be made compulsory, and it is the obvious duty of the new Minister of Health to press upon his colleagues the necessity of returning to the original rigidity of the law which was so unwisely modified some years ago. There would be an outcry, of course, but it is the duty of Governments to ignore outcries when they have every justification for believing that they are taking the right course in the public interest. But the spectre of the ballot-box haunts the pillow of every politician, since the first article of his creed is the paramount importance of his own preservation. Yet modern Governments might sometimes try boldness by way of a change. It has quite often been known to succeed.

### TOO DULL OR TOO CLEVER?

MAN, said Dr. Cyril Burt, in his presidential address to the Psychology Section of the British Association, is "something more than a carcass loosely coupled with a ghost." The epigram, like all other brilliant epigrams, contains a truth so obvious that we constantly lose sight of it. His subject was "The Mental Differences between Individuals," and he did not lose the opportunity of jumping from epigram to paradox. It is, he declares, a blunder always to choose the brightest candidate for a given job—he may be much too clever for it. But, while that might be a disadvantage to the particular job, it would probably, in the end, not be a disadvantage to its holder, who would naturally proceed to something which presented a wider scope for his abilities. Every able young man eventually becomes "too clever" for his original task and, with ordinary good fortune, is called up higher. On the other hand, as we all know, many jobs are performed by people who are too dull. Dr. Burt was on stronger ground when he laid stress upon the close relation between physical and moral health. Exceptionally large adenoids, septic tonsils, decayed teeth, or defective sight, may easily account for a tendency to petty larceny or even graver offences, and the removal of these unhealthy conditions or the provision of spectacles has often converted an apparently mentally defective child into a normal, or nearly normal, being. We have only lately begun to realise the extreme intimacy of the tie between body and mind. Something has always been known of it, but that mental twists may be cured by surgery we have begun fully to understand only of late. It is a sociological fact of the first importance, and he would be a bold, not to say a rash, man who should prophesy whither, in the course of the next generation or two, it may lead us.

### SIR WILLIAM TRELOAR.

ONLY six months ago when, in our jovial English way, Sir William Treloar's philanthropic work was recognised by a luncheon at the Mansion House, we said that few Lord Mayors of London had created so splendid a memorial of their year of office, and now that we lament to record his death, we are unable to better the phrase. The allusion was, of course, to his creation of the Cripples' Hospital at Alton, in Hampshire, with the £60,000 which he collected by a Mansion House Fund. That is sixteen years ago, and for half a generation that most beneficent hospital has been helping unhappy children to get the better of the handicaps imposed upon them by tuberculosis. Not only has it produced physical amelioration which would otherwise have been impossible, but it has equipped many a lad with a trade that has enabled him to earn his living as an independent member of the community. Not so long ago his lot would have been pitiable indeed. By his wise thoughtfulness Sir William Treloar substantially reduced the misery worked by a dread disease which is even now not under control, and his death leaves a wide gap in the ranks of Good Samaritans. But the example and influence of such men does not die, and others will be inspired to continue and enlarge this and similar work.

### THE PURITY OF FOOD.

THE daily newspapers always, in August and September, touchingly solicitous for the public health, have been giving a good deal of space to the adulteration of food and the careless way in which it is handled. That many alimentary articles are far from pure and that the law in regard to them needs strengthening is unquestioned, but it will be of little avail to insist upon a high standard of internal purity so long as we allow meat, fish, bread and innumerable other foods to be carelessly contaminated on their way to the consumer's table. Dust blows upon them, dirty fingers handle them, flies settle upon them, they are tumbled into unclean baskets, and dealt with as if they were of no greater moment than tennis balls. Thus we have to deal with original and acquired impurity, and the work is not made the easier by the comparative indifference and the positive ignorance of the general public. Most people do not know the difference between good pure food and sophisticated or inferior food—if they did they would not for a week endure the grotesque product called "bread," which is accountable for a large proportion of the national indigestion. It is only by "pegging away" at subjects of this kind that reforms are brought about, but meanwhile the standard that has recently been established for condensed milk is an earnest that the Ministry of Health is by no means quiescent.

### THE PROBLEM OF THE DIPHTHERIA CARRIER.

INVESTIGATIONS recently carried out in a Danish fever hospital show that it is most difficult to be sure that a child who has suffered from diphtheria is no longer infectious on discharge from hospital. It is a common experience that the frequency of "return" cases of diphtheria is as great after patients have been discharged as non-infectious as when they have been discharged with a positive bacteriological report. The truth of the matter would seem to be that one or two negative bacteriological examinations are of very little value; only when five consecutive examinations have proved negative are there reasonable grounds for believing that the patient is no longer infectious. But there may be as many as seven consecutive negative examinations, and yet the eighth examination may reveal virulent diphtheria bacilli. Among 100 candidates for discharge from diphtheria wards there were 40 who obtained three consecutive negative bacteriological reports, and who at a later examination were still found to harbour virulent diphtheria bacilli. To keep all such cases isolated till they can pass the most stringent bacteriological tests would mean adding enormously to the cost of running a fever hospital, and in Denmark, at any rate, specialists in this sphere seem to have resigned themselves to discharging from hospital diphtheria convalescents an unknown number of whom are probably still infectious. They appear to hope for salvation, not from strict isolation of every diphtheria carrier, but from a wholesale application of Schick's test, which indicates whether or not the tested person is susceptible to diphtheria. By vaccinating according to Behring's method all susceptible persons, it is anticipated that far more effective control of diphtheria will be achieved than by the older and more primitive method of isolation.

### PEOPLING THE DOMINIONS.

A VERY suggestive address advocating the maintenance of the birth-rate was delivered by Dr. Vaughan Cornish, one of our most distinguished geographers, in his presidential remarks to the Geographical Section of the British Association. Dr. Cornish's point is that the Dominions are at present unable, by their own natural increase of population, to do much towards peopling their vast waste spaces, and that, since it is of primary importance that those spaces should be peopled by British stocks, it is our duty to help them out. The birth-rate of Great Britain should therefore be maintained above the death-rate at least until the British population in the Dominions exceeds that in the mother country. There is no immediate probability of our birth-rate falling below that of deaths, yet the process of peopling great Continents is slow and by the time Dr. Cornish's requirement is met, the ratio may well have undergone serious alteration. However widely opinions upon birth-control may vary, it is impossible to call in question Dr. Cornish's dictum that unless the British race increases we cannot insure the internal peace and external security of the Empire, or the continuance of its beneficent work of enlarging commerce and restricting the range of war. At present our home population grows far more rapidly than that of the Dominions, even allowing for emigration. This being so, it is the more regrettable that the new Government scheme for emigration has, so far, been much less successful than had been hoped. We can well spare some of the population of which the Dominions are in sore need.

### MEDICAL TESTS FOR DRUNKEN DRIVERS.

ROAD-HOGS may be classified according as they are sober or drunk. Not that there is much difference; even when he is "dry," the road-hog is a noisome pest. But the drunken hog being even more pestilential, there is a movement on foot in this country to deprive him of his driver's licence for ever. It is interesting to note that this rule has already been adopted in Denmark where, since 1921, a new law has been put into force permanently robbing the motor driver of his licence after a conviction for drunkenness. This salutary reform has led to a revision of the customary tests for drunkenness, and in order that there shall be no miscarriage of justice, the Danish police have co-operated with the Medico-Legal Council in Copenhagen with a view to adopting a uniform system for investigating alleged cases of drunkenness. The drunken driver, put through these tests, must, if he is not too drunk, feel like a camel confronted with the eye of a needle. The examination is carried out in a room occupied only by the police surgeon and the driver, and as the tests take, on an average, about forty-five minutes, the bodily and mental capacity of the alleged drunkard is exhaustively investigated. He may, for a few moments, pull himself together in the manner familiar to most of us, but to keep alert for nearly an hour, solving arithmetical problems, reading aloud from a newspaper, and giving a lucid and consecutive account of recent events, is a task requiring the driest of dry sobriety. Recent experience has shown that counting the pulse and getting the driver to write his name are useless tests, and were we to judge of the sobriety of our fellows by their signatures, it is to be feared that few would escape classification as heavy drinkers. But, on the whole, the Danish system of medical examinations seems to be working satisfactorily. This process is one which might well be copied in this country; to let a drunken driver off with a fine or a politely-endorsed licence is, in many cases, equivalent to being accessory before the event to a murder.

### THE BLOOD-PRESSURE HYPOCHONDRIAC.

A NEW crop of hypochondriacs has recently erupted in this highly civilised world. They are the product of the modern and still very immature teaching that the blood-pressure is a trustworthy guide to the health of the individual. Time was when our health was gauged by cardiac murmurs, and many a perfectly healthy person spent his life, from adolescence to querulous old age, wondering all the time when the thin thread of his life would snap and that murmur, to which a score of eminent physicians had testified, would be silenced for ever. Had he but known that the sword of Damocles was suspended by a steel cable and not by a thread, his life might have been useful instead of a nuisance. The work of Sir James McKenzie and others has brought it home even to the most obscure Harley Street physician that many, if not most, cardiac murmurs have no prognostic significance, and the "murmur-hypochondriac" is becoming a rare bird. Unfortunately, the "blood-pressure hypochondriac" is becoming very common. He or she wanders from one specialist to another, being plunged into the depths of despair when the blood-pressure is found a few millimetres higher than before, and rejoicing as if a rich uncle had died when the blood-pressure shows a slight fall. The medical officer of a large hydro in Germany has recently drawn attention to this nuisance, which is cosmopolitan. He describes blood-pressure measurements as a psychic trauma, and he refers to the case of an able-bodied man who declined a very lucrative and important appointment because, forsooth, he feared his blood-pressure was not equal to the strain. It is well for patients to know that fear alone may raise the blood-pressure from 100 to 150 millimetres of mercury, and that hypnotism may reduce it from 200 to 155. Patients who are informed that they have a high blood-pressure and that their lives are threatened would be well advised if they accepted this verdict with reserve, remembering that in many cases the secret of long life is to suffer from some so-called incurable ailment.

### THE OMNIPOTENT THYROID GLAND.

THE thyroid gland is much in the limelight at present. It has long been known that certain forms of idiocy are due to defective action of the thyroid, and that giving thyroid extract by the mouth may in such cases convert an idiot into a perfectly sane member of society. Quite recently several other disorders have been traced to the delinquent thyroid, and a doctor in Dresden has lately cured a patient suffering from Dercum's disease, which is characterised by painful fatty tumours, general muscular weakness and psychic disorders. Giving thyroid extract by the mouth converted this patient, a woman aged forty-seven, from a hypochondriacal invalid into an able-bodied and mentally normal creature. Another German doctor traces pyorrhœa alveolaris to faulty action of the glands of internal secretion, among which the thymus and thyroid would seem to be arch offenders. The various germs found in the gums in cases of pyorrhœa are, in his opinion, of secondary importance, and they would probably do little harm were not the tissues predisposed to pyorrhœa by the constitutional weakness traceable to faulty action of the thymus, thyroid and their confederates. A Swiss doctor has lately been most successful in treating itching or *pruritus genitalis* with dried thyroid substance given by the mouth after various local remedies had been given a fair trial and had failed. Doubtless many other complaints of mind and body will, in the near future, be traced to the faulty action of the thyroid, and we may perhaps look forward to the time when wars and other troubles will be no more, because the passions generating them have been soothed by a course of thyroid medication given by the League of Nations to obstreperous politicians and prime ministers who hurl angry ultimatums at each other for want of a little thyroid extract.

### HEALTHIER WOMANHOOD.

THE increasingly strenuous life led by the modern woman would appear to have a distinctly beneficial effect upon her general well-being. Dr Campbell, of Guy's Hospital, has been commenting upon the rapid disappearance from among young girls of anæmia, or what was once called the "green sickness." This he attributes very largely to their abundant opportunities for fresh air and exercise. It would seem indeed that the general health of womanhood in all civilised countries is steadily improving—an improvement which began in the early years of this century, when athletics first became really popular among girls. There can be no doubt, moreover, that women's wider mental activities have distracted them from the consideration of their own health, and the girl of to-day is as anxious to conceal any passing indisposition as her great-grandmother was to display it. Dr. Christine Murrell, in *Womanhood and Health*, which has just been published by Messrs. Mills and Boon, thinks that the modern style of feminine dress does much to make for ease, health and efficiency. She takes comfort from the fact that "the designers of fashions no longer have such a docile crowd of followers as formerly," and points out that the "only articles of apparel in which women are at the present time encouraged to place the body in a position mechanically abnormal are their boots and shoes." This is a great advance on the fashion of twenty years ago, which may have helped woman to be ornamental but hindered her from being useful. Dr. Murrell suggests that the "increasing magnitude of woman's share in the care and nourishment of the new generation as civilisation advanced caused a change in the general attitude of men towards her from that of comrade to one composed of a curious mixture of wonder, reverence and pity." That is an attitude which Dr. Murrell and most other women want to change. Women are in some respects stronger physically than men, and as they develop their physical and mental powers to an extent undreamt of fifty years ago, so can they most satisfactorily fulfil their new duties as citizens and their older duties as wives and mothers.

### THE DREYER TUBERCULOSIS VACCINE.

IT will be remembered that after Professor Dreyer's preliminary announcement a few months ago, a certain number of physicians and pathologists undertook to investigate the action of this vaccine on patients suffering from tuberculosis and on guinea-pigs experimentally infected with the tubercle bacillus. These investigations are still proceeding, and it is probable that some time will yet elapse before official reports thereon can be published. It is to be feared, however, that the high hopes entertained with regard to this remedy are unlikely to be justified—neither the tuberculous patient nor the experimental animal has responded as satisfactorily as had been expected. There have, of course, been isolated instances of improvement under this treatment, but the uniform betterment so hopefully anticipated after Professor Dreyer's first publication has not been demonstrated. So far the matter is clear. It is, however, at present complicated by the fact that some of the investigators who have undertaken to test this remedy consider quite naturally that, in common fairness to Professor Dreyer, the material they have collected and the experience they have gained should be pooled, so that, in the fullness of time, an exhaustive report may be published by Professor Dreyer and his associates. Meantime, thousands of patients and their relatives are anxiously waiting for confirmation of the earlier claims made on behalf of this remedy. Between success and failure there may, as we pointed out in an earlier issue, be only a small gap, and the diaplyte vaccine may yet prove beneficial after certain modifications have been introduced in the technique of its administration. But it should at once be made clear that, as it is being given to-day, it is not that elixir of life which many of us hoped it might prove to be.

